# Supplementary material for: Collagen I Promotes Adipocytogenesis in Adipose-Derived Stem Cells In Vitro
Source: Cells. 2019 Apr 1;8(4):302. doi: 10.3390/cells8040302 (PMC6523348; doi:10.3390/cells8040302)
Supplement: Supplementary file 1 [file cells-08-00302-s001.pdf]

Fig. S1

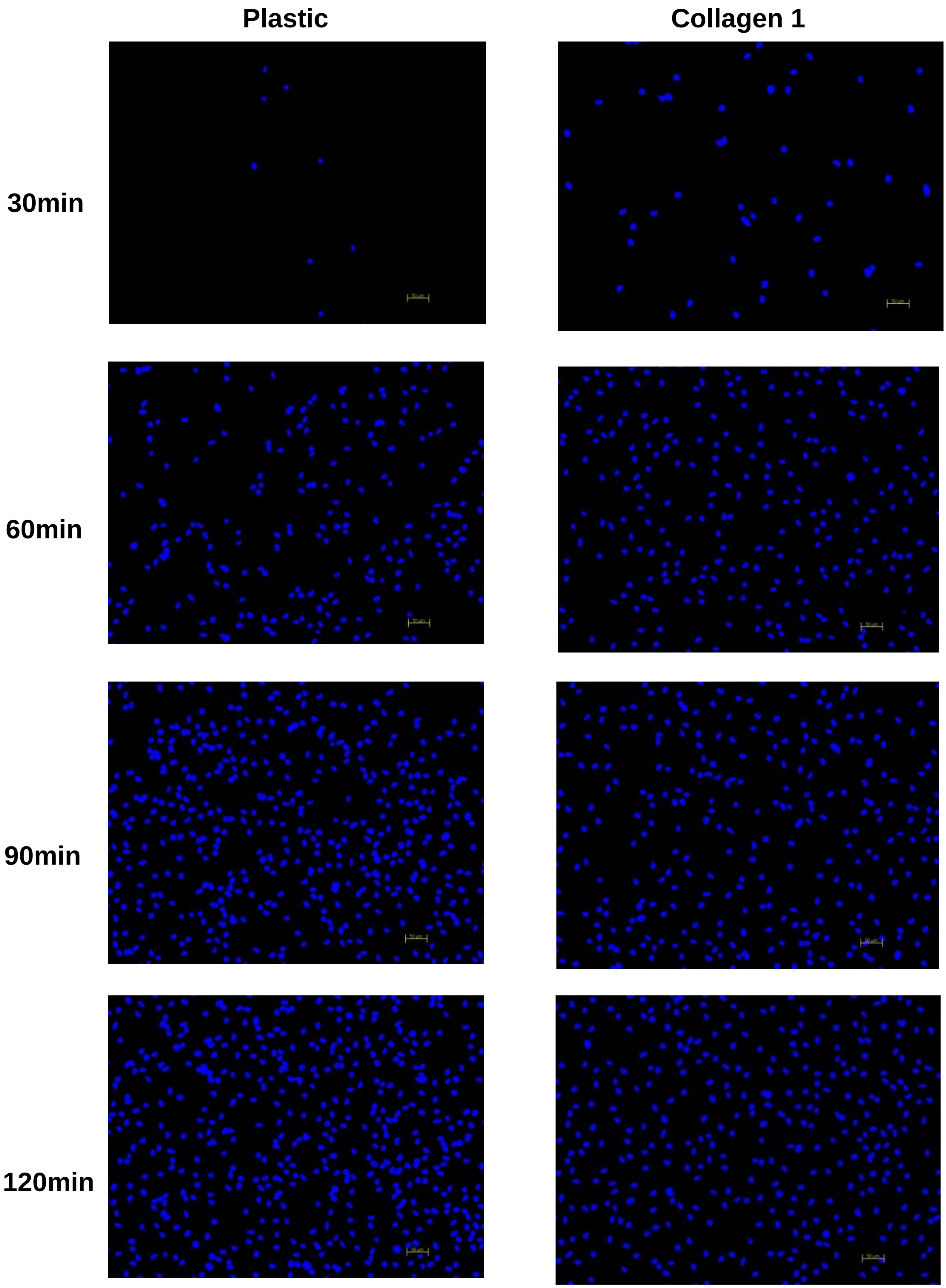

Fig. S1: Time-dependent cell adhesion of ASCs to plastic vs collagen I. ASCs were plated at a density of $1x10^4$ cells per well into microtiterplates either non-coated or coated with collagen I. After 30, 60, 90 and 120 min non-adherent cells were discarded and the remaining cells were stained with bisbenzimide. The photographs show representative sections
